# Development of an inactivated vaccine candidate for SARS-CoV-2

**DOI:** 10.1126/science.abc1932

**Published:** 2020-05-06

**Authors:** Qiang Gao, Linlin Bao, Haiyan Mao, Lin Wang, Kangwei Xu, Minnan Yang, Yajing Li, Ling Zhu, Nan Wang, Zhe Lv, Hong Gao, Xiaoqin Ge, Biao Kan, Yaling Hu, Jiangning Liu, Fang Cai, Deyu Jiang, Yanhui Yin, Chengfeng Qin, Jing Li, Xuejie Gong, Xiuyu Lou, Wen Shi, Dongdong Wu, Hengming Zhang, Lang Zhu, Wei Deng, Yurong Li, Jinxing Lu, Changgui Li, Xiangxi Wang, Weidong Yin, Yanjun Zhang, Chuan Qin

**Affiliations:** 1 Sinovac Biotech Ltd., Beijing, China.; 2 Key Laboratory of Human Disease Comparative Medicine, Chinese Ministry of Health, Beijing Key Laboratory for Animal Models of Emerging and Remerging Infectious Diseases, Institute of Laboratory Animal Science, Chinese Academy of Medical Sciences and Comparative Medicine Center, Peking Union Medical College, Beijing, China.; 3 Department of Microbiology, Zhejiang Provincial Center for Disease Control and Prevention, Hangzhou, China.; 4 Division of Respiratory Virus Vaccines, National Institute for Food and Drug Control, Beijing, China.; 5 CAS Key Laboratory of Infection and Immunity, National Laboratory of Macromolecules, Institute of Biophysics, Chinese Academy of Sciences, Beijing, China.; 6 National Institute for Communicable Disease Control and Prevention, Chinese Center for Disease Control and Prevention, Changping, Beijing, China.; 7 Institute of Microbiology and Epidemiology, Academy of Military Medical Sciences, Beijing, China.

## Abstract

The coronavirus disease 2019 (COVID-19) pandemic caused by severe acute respiratory syndrome–coronavirus 2 (SARS-CoV-2) has resulted in an unprecedented public health crisis. There are currently no SARS-CoV-2-specific treatments or vaccines available due to the novelty of the virus. Hence, rapid development of effective vaccines against SARS-CoV-2 are urgently needed. Here we developed a pilot-scale production of a purified inactivated SARS-CoV-2 virus vaccine candidate (PiCoVacc), which induced SARS-CoV-2-specific neutralizing antibodies in mice, rats and non-human primates. These antibodies neutralized 10 representative SARS-CoV-2 strains, suggesting a possible broader neutralizing ability against SARS-CoV-2 strains. Three immunizations using two different doses (3 μg or 6 μg per dose) provided partial or complete protection in macaques against SARS-CoV-2 challenge, respectively, without observable antibody-dependent enhancement of infection. These data support clinical development of SARS-CoV-2 vaccines for humans.

The World Health Organization declared the outbreak of coronavirus disease in 2019 (COVID-19) to be a Public Health Emergency of International Concern on 30 January 2020, and a pandemic on 11 March 2020. It is reported that ~80% of COVID-19 patients have mild-to-moderate symptoms, while ~20% develop serious manifestations such as severe pneumonia, acute respiratory distress syndrome (ARDS), sepsis and even death (*1*). The number of COVID-19 cases has increased at a staggering rate globally. Severe acute respiratory syndrome–coronavirus 2 (SARS-CoV-2), the causative virus of the ongoing pandemic, belongs to the genus *Betacoronavirus* (β-CoV) of the family *Coronavirdae* (*2*). SARS-CoV-2 along with the severe acute respiratory syndrome coronavirus (SARS-CoV) and the Middle Eastern respiratory syndrome-related coronavirus (MERS-CoV), constitute the three most life-threatening species among all human coronaviruses. SARS-CoV-2 harbors a linear single-stranded positive sense RNA genome, encoding 4 structural proteins [spike (S), envelope (E), membrane (M), and nucleocapsid (N)] of which S is a major protective antigen that elicits highly potent neutralizing antibodies (NAbs), 16 non-structural proteins (nsp1-nsp16) and several accessory proteins (*3*). No specific antiviral drugs or vaccines against the newly emerged SARS-CoV-2 are currently available. Therefore, urgency in the development of vaccines is of vital importance to curb the pandemic and prevent new viral outbreaks.

Multiple SARS-CoV-2 vaccine types, such as DNA-, RNA-based formulations, recombinant-subunits containing viral epitopes, adenovirus-based vectors and purified inactivated virus are under development (*4*–*6*). Purified inactivated viruses have been traditionally used for vaccine development and such vaccines have been found to be safe and effective for the prevention of diseases caused by viruses like influenza virus and poliovirus (*7*, *8*). To develop preclinical in vitro neutralization and challenge models for a candidate SARS-CoV-2 vaccine, we isolated SARS-CoV-2 strains from the bronchoalveolar lavage fluid (BALF) samples of 11 hospitalized patients (including 5 patients in intensive care), among which 5 are from China, 3 from Italy, 1 from Switzerland, 1 from UK and 1 from Spain (table S1). These patients were infected with SARS-CoV-2 during the most recent outbreak. The 11 samples contained SARS-CoV-2 strains that are widely scattered on the phylogenic tree constructed from all available sequences, representing, to some extent, circulating SARS-CoV-2 populations (Fig. 1A and fig. S1). We chose strain CN2 for purified inactivated SARS-CoV-2 virus vaccine development (PiCoVacc) and another 10 strains (termed as CN1, CN3-CN5 and OS1-OS6) as preclinical challenge strains. Of note, the CN1 and OS1 strains are closely related to 2019-nCoV-BetaCoV /Wuhan/WIV04/2019 and EPI_ISL_412973, respectively, which have been reported to cause severe clinical symptoms, including respiratory failure, requiring mechanical ventilation (*9*, *10*).

**Fig. 1:**
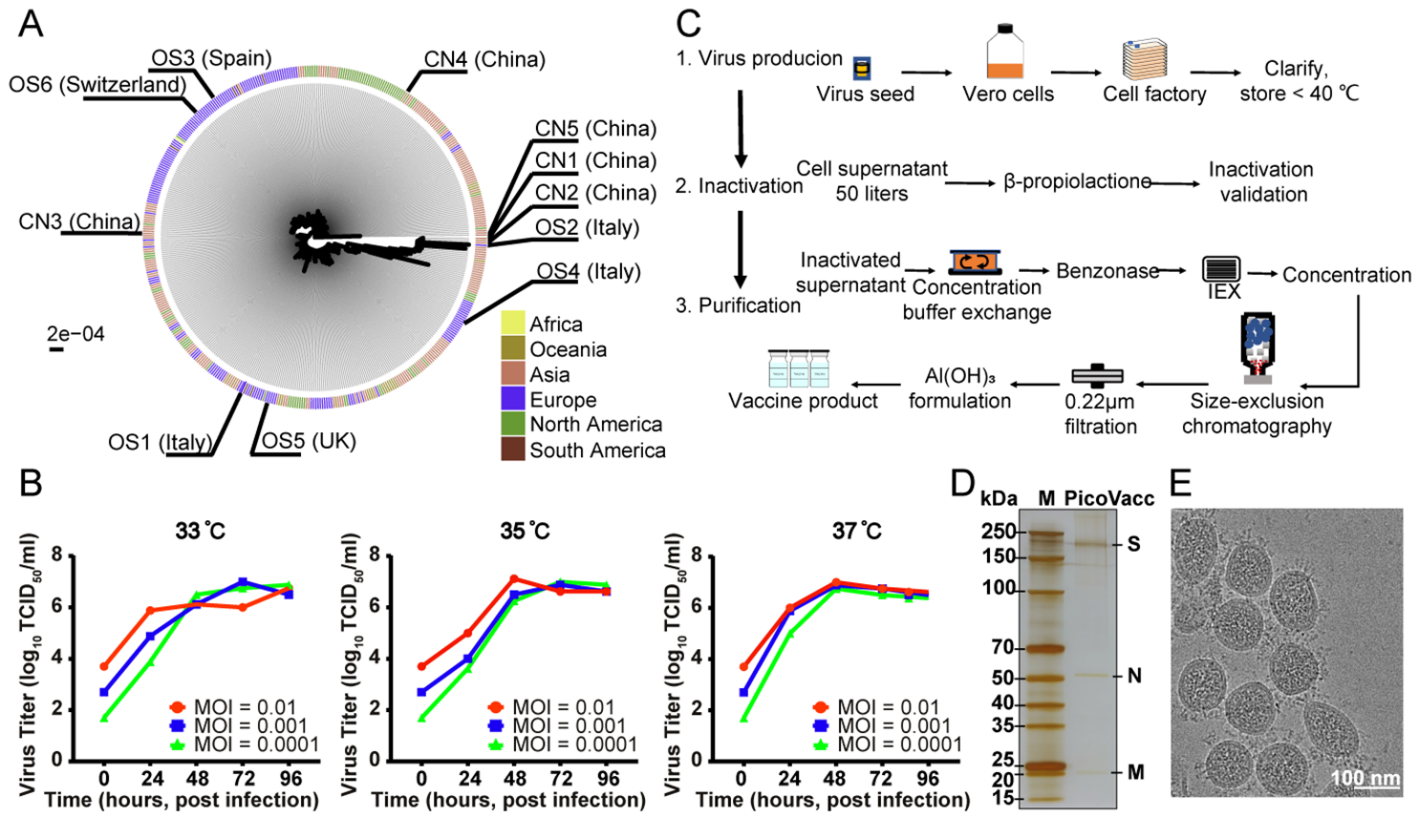
Characterization of the SARS-CoV-2 vaccine candidate, PiCoVacc. (**A**) The SARS-CoV-2 maximum likelihood phylogenetic tree. The SARS-CoV-2 isolates used in this study are depicted with black lines and labeled. Viral strains were isolated in infected patients who traveled from the continents as indicated (**B**) Growth kinetics of PiCoVacc (CN2) P5 stock in Vero cells. (**C**) Flowchart of PiCoVacc preparation. (**D**) Protein composition and purity evaluation of PiCoVacc by NuPAGE 4-12% Bis-Tris Gel. (**E**) Representative electron micrograph of PiCoVacc. White scale bar = 100 nm.

To obtain a viral stock adapted for efficient growth in Vero cells for PiCoVacc production, the CN2 strain was first plaque purified and passaged once in Vero cells to generate the P1 stock. After this another four passages were performed to generate the P2-P5 stocks. Growth kinetics analysis of the P5 stock in Vero cells showed that this stock replicated efficiently and reached a peak titer of 6-7 log_10_ TCID_50_/ml by 3 or 4 days post infection (dpi) at multiplicities of infection (MOI) of 0.0001-0.01 at temperatures between 33°C-37°C (Fig. 1B). To evaluate the genetic stability of PiCoVacc, 5 more passages were performed to obtain the P10 stock, whole genome of which, together with those of the P1, P3 and P5 stocks were sequenced. Compared to P1, only two amino acid substitutions, Ala → Asp at E residue 32 (E-A32D) and Thr → Ile at nsp10 residue 49 (nsp10-T49I), occurred in P5 and P10 stocks (table S2), suggesting that PiCoVacc CN2 strain possesses excellent genetic stability without the S mutations that might potentially alter the NAb epitopes. To produce pilot scale PiCoVacc for animal studies, the virus was propagated in a 50-liter culture of Vero cells using the Cell Factory system and inactivated by using β-propiolactone (Fig. 1C). The virus was purified using depth filtration and two optimized steps of chromatography, yielding a highly pure preparation of PiCoVacc (Fig. 1D). Additionally, cryo-electron microscopy (cryo-EM) analysis showed intact oval-shaped particles with diameters of 90-150 nm, which are embellished with crown-like spikes, representing a prefusion state of the virus (Fig. 1E).

To assess the immunogenicity of PiCoVacc, groups of BALB/c mice (n=10) were injected at day 0 and day 7 with various doses of PiCoVacc mixed with alum adjuvant (0, 1.5 or 3 or 6 μg per dose, 0 μg in physiological saline as the sham group). No inflammation or other adverse effects were observed. Spike-, receptor binding domain (RBD)-, and N-specific antibody responses were evaluated by enzyme-linked immunosorbent assays (ELISAs) at weeks 1-6 after initial immunization (fig. S2). SARS-CoV-2 S- and RBD-specific immunoglobulin G (Ig G) developed quickly in the serum of vaccinated mice and peaked at the titer of 819,200 (>200 μg/ml) and 409,600 (>100 μg/ml), respectively, at week 6 (Fig. 2A). RBD-specific IgG accounts for half of the S-induced antibody responses, suggesting RBD is the dominant immunogen, which closely matches the serological profile of the blood of recovered COVID-19 patients (Fig. 2, A and B) (*11*). Surprisingly, the amount of N-specific IgG induced is ~30-fold lower than the antibodies targeting S or RBD in immunized mice (Fig. 2A). Interestingly, previous studies have shown that the N-specific IgG is largely abundant in the serum of COVID-19 patients and serves as one of the clinical diagnostic markers (*11*). It’s worthy to note that PiCoVacc could elicit ~10-fold higher S-specific antibody titers in mice than those of the serum from the recovered COVID-19 patients (Fig. 2, A and B). Although this observation is currently not indicative of PiCoVacc’s ability to produce similar results in humans, it highlights the potential of PiCoVacc to induce a strong and potent immune response. Taken together, our findings - coupled with the fact that the antibodies targeting N of SARS-CoV-2 do not provide protective immunity against the infection (*12*) - suggest that PiCoVacc might be capable of eliciting more effective antibody responses (Fig. 2, A and B).

**Fig. 2:**
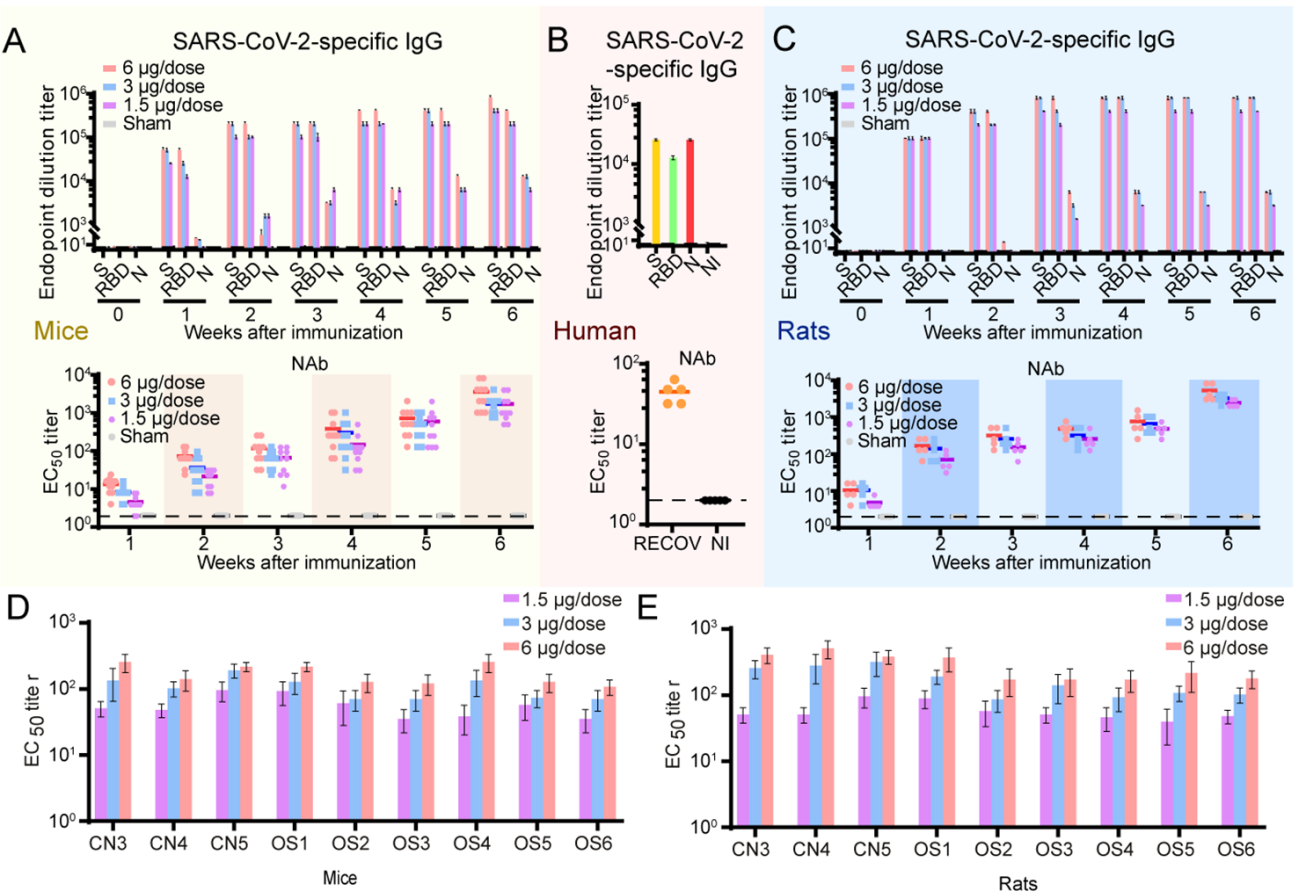
PiCoVacc immunization elicits neutralizing antibody response against ten representative SARS-CoV-2 isolates. BALB/c mice and Wistar rats were immunized with various doses of PiCoVacc or control (adjuvant only as the sham group) (n=10). Serums from recovered COVID19 patients (RECOV) and non-infected (NI) individuals were used as positive and negative controls, respectively. The antibody responses were analyzed in mice (**A**), humans (**B**) and rats (**C**). Top: SARS-CoV-2-specific IgG responses as measured by ELISA; bottom: neutralizing antibody titer determined by microneutralization assay. The spectrum of neutralizing activities elicited by PiCoVacc was investigated in mice (**D**) and rats (**E**). Neutralization assays against the other nine isolated SARS-CoV-2 strains was performed using mouse and rat serums collected 3 weeks post-vaccination. Data points represent mean +/− SEM of individual animals and humans from five to ten independent experiments; error bars reflect SEM;; dotted lines indicate the limit of detection; horizontal lines indicate the geometric mean titer (GMT) of EC_50_ for each group.

Next, we measured SARS-CoV-2-specific neutralizing antibodies over a period of time using microneutralization assays (MN50). Similar to S-specific IgG responses, the neutralizing antibody titer against the CN1 strain emerged at week 1 (12 for high dose immunization), surged after the week 2 booster and reached up to around 1,500 for low and medium doses, and 3,000 for the high dose at week 7, respectively (Fig. 2A). In contrast, the sham group did not develop detectable SARS-CoV-2-specific antibody responses (Fig. 2, A and B). In addition, immunogenic evaluations of PiCoVacc in Wistar rats with the same immunization strategy yielded similar results - the maximum neutralizing titers reached 2,048-4,096 at week 7 (Fig. 2C). To investigate the spectrum of neutralizing activities elicited by PiCoVacc, we conducted neutralization assays against the other 9 isolated SARS-CoV-2 strains using mouse and rat serums collected 3 weeks post vaccination. Neutralizing titers against these strains demonstrate that PiCoVacc is capable of eliciting antibodies that possibly exhibit potent neutralization activities against SARS-Cov-2 strains circulating worldwide (Fig. 2, D and E).

We next evaluated the immunogenicity and protective efficacy of PiCoVacc in rhesus macaques (*Macaca mulatta*), a non-human primate species that shows a COVID-19-like disease caused by SARS-CoV-2 infection (*13*). Macaques were immunized three times via the intramuscular route with medium (3 μg per dose) or high doses (6 μg per dose) of PiCoVacc at day 0, 7 and 14 (n=4). S-specific IgG and NAb were induced at week 2 and rose to ~12,800 and ~50, respectively at week 3 (before virus challenge) in both vaccinated groups, whose titers are similar to those of serum from the recovered COVID-19 patients (Fig. 3, A and B). Unexpectedly, NAb titer (61) in the medium dose immunized group were ~20% greater than that observed (50) in the high dose vaccinated group at week 3, possibly due to individual differences in the ability of one animal in the medium dose group in eliciting ~10-fold higher titer when compared to the other three animals (Fig. 3B). Excluding this exception, NAb titer in the medium dose group would drop down to 34, ~40% lower than that in the high dose group. Subsequently, we conducted a challenge study by a direct inoculation of 10^6^ TCID_50_ of SARS-CoV-2 CN1 into the animal lung through the intratracheal route at day 22 (one week after the third immunization) in vaccinated and control macaques to verify the protective efficacy. Expectedly, all control (sham and placebo) macaques showed excessive copies (10^4^-10^6^/ml) of viral genomic RNA in the pharynx, crissum and lung by day 3-7 post-inoculation (dpi) and severe interstitial pneumonia (Fig. 3, C to F). By contrast, all vaccinated macaques were largely protected against SARS-CoV-2 infection with very mild and focal histopathological changes in a few lobes of lung, which probably were caused by a direct inoculation of 10^6^ TCID_50_ of virus into the lung through intratracheal route, that needed longer time (more than one week) to recover completely (Fig. 3F). Viral loads decreased significantly in all vaccinated macaques, but increased slightly in control animals from day 3-7 after infection (Fig. 3, C to E). All four macaques that received the high dose, had no detectable viral loads in pharynx, crissum and lung at day 7 after infection. In the medium dose immunized group, we indeed partially detected the viral blip from pharyngeal (3/4), anal (2/4) and pulmonary (1/4) specimens at day 7 after infection, while viral loads presented a ~95% reduction when compared to the sham groups (Fig. 3, C to E). Interestingly, NAb titer in vaccinated groups decreased by ~30% by 3 days post infection to neutralize viruses, then rapidly increased from day 5-7 after infection to maintain neutralization efficacy. In comparison with high dose vaccination group (titer of ~145), higher NAb titers observed in the medium dose vaccinated group at day 7 after infection (~400 for 4 macaques) might have resulted from relatively low levels of viral replication, suggesting a requirement of longer time for complete viral clearance. No antibody-dependent enhancement (ADE) of infection was observed for the vaccinated macaques despite the observation that relatively low NAb titer existed within the medium dose group before infection, offering partial protection. The possibility of manifestation of ADE after antibody titers wane could not be ruled out in this study. Further studies involving observation of challenged animals at longer periods of time post vaccination are warranted to address this.

**Fig. 3:**
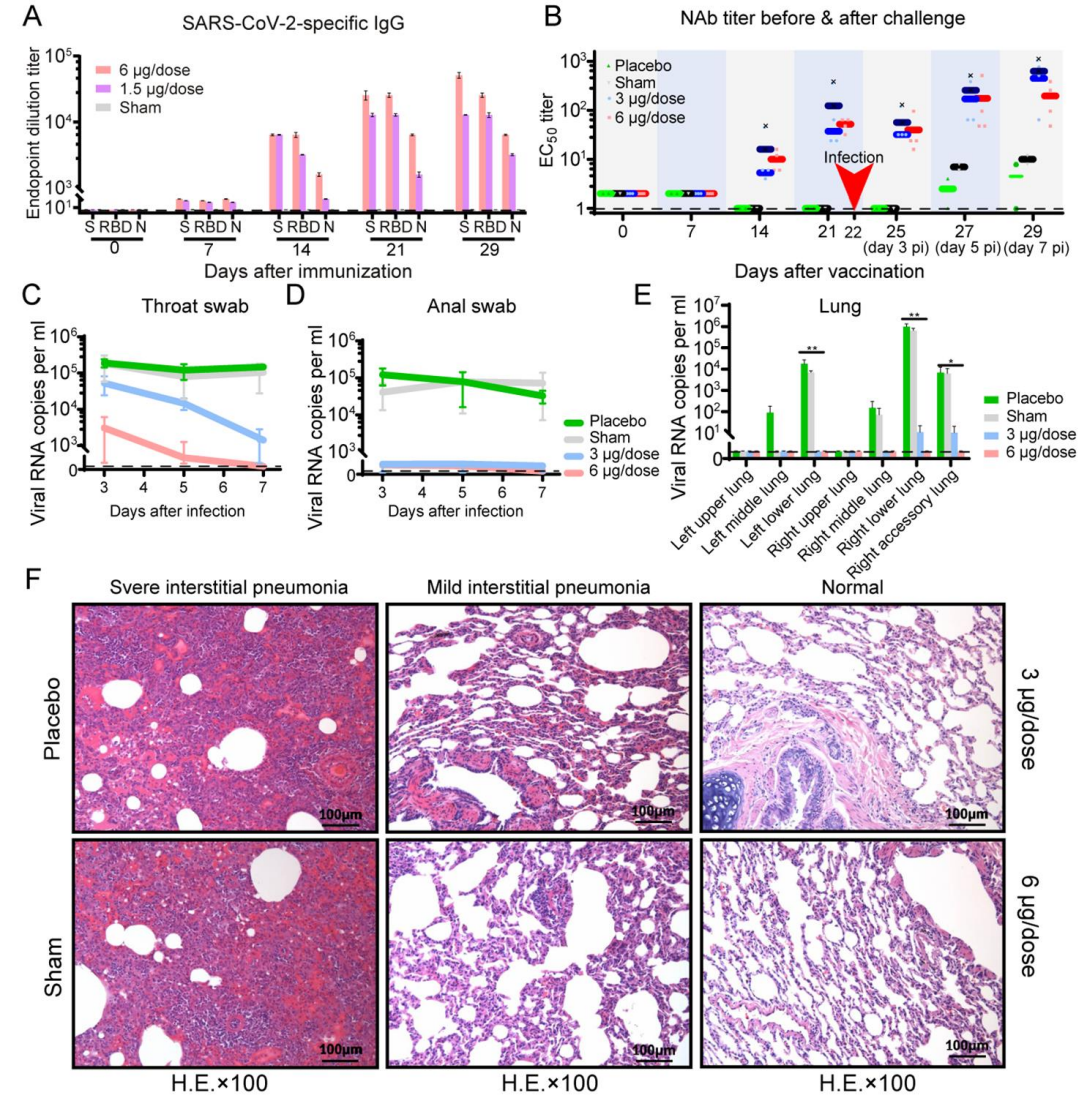
Immunogenicity and protective efficacy of PiCoVacc in nonhuman primates. Macaques were immunized three times through the intramuscular route with various doses of PiCoVacc or adjuvant only (sham) or placebo (n=4). SARS-CoV-2-specific IgG response (**A**) and neutralizing antibody titer (**B**) were measured. Data points represent mean +/− SEM of individual macaques from four independent experiments; error bars reflect SEM; dotted lines indicate the limit of detection; horizontal lines indicate the geometric mean titer (GMT) of EC_50_ for each group. Protective efficacy of PiCoVacc against SARS-CoV-2 challenge at week 3 after immunization was evaluated in macaques (**C-F**). Viral loads of throat (**C**) and anal (**D**) swab specimens collected from the inoculated macaques at day 3, 5 and 7 pi were monitored. Viral loads in various lobes of lung tissue from all the inoculated macaques at day 7 post-infection were measured (**E**). RNA was extracted and viral load was determined by qRT-PCR. All data are presented as mean ± SEM from four independent experiments; error bars reflect SEM. Asterisks represent significance: *P < 0.05 and **P < 0.01. Histopathological examinations (**F**) in lungs from all the inoculated macaques at day 7 post infection. Lung tissue was collected and stained with hematoxylin and eosin.

Previous reports on the development of SARS and MERS vaccine candidates raised concerns about pulmonary immunopathology, either directly caused by a type 2 helper T-cell (Th2) response or as a result of ADE (*4*, *14*, *15*). Although T-cell responses elicited by many vaccines have been demonstrated to be crucial for acute viral clearance, protection from subsequent coronavirus infections is largely mediated by humoral immunity (*16*, *17*). The “cytokine storm” induced by excessive T-cell responses have been actually shown to accentuate the pathogenesis of COVID19 (*18*, *19*). Therefore, T-cell responses elicited by any SARS-CoV-2 vaccine(s) would have to be well controlled in order to avoid immunopathology. In this context, we systematically evaluated safety of PiCoVacc in macaques by recording a number of clinical observations and biological indices. Two groups of macaques (n=10) were immunized by intramuscular injection with low (1.5 μg) or high doses (6 μg) and another two groups of macaques (n=10) were immunized with adjuvant (sham) and physiological saline (placebo) for three times at day 0, 7 and 14 time points. Neither fever nor weight loss was observed in any macaque after the immunization of PiCoVacc, and the appetite and mental state of all animals remained normal (fig. S3). Hematological and biochemical analysis, including biochemical blood test, lymphocyte subset percent (CD3^+^, CD4^+^ and CD8^+^) and key cytokines (TNF-α, IFN-γ, IL-2, IL-4, IL-5 and IL-6) showed no notable changes in vaccinated groups when compared to the sham and placebo groups (Fig. 4, A and B, and figs. S4 and S5). In addition, histopathological evaluations of various organs, including lung, heart, spleen, liver, kidney and brain, from the 4 groups at day 29 demonstrated that PiCoVacc did not cause any notable pathology in macaques (Fig. 4C and fig. S6).

**Fig. 4:**
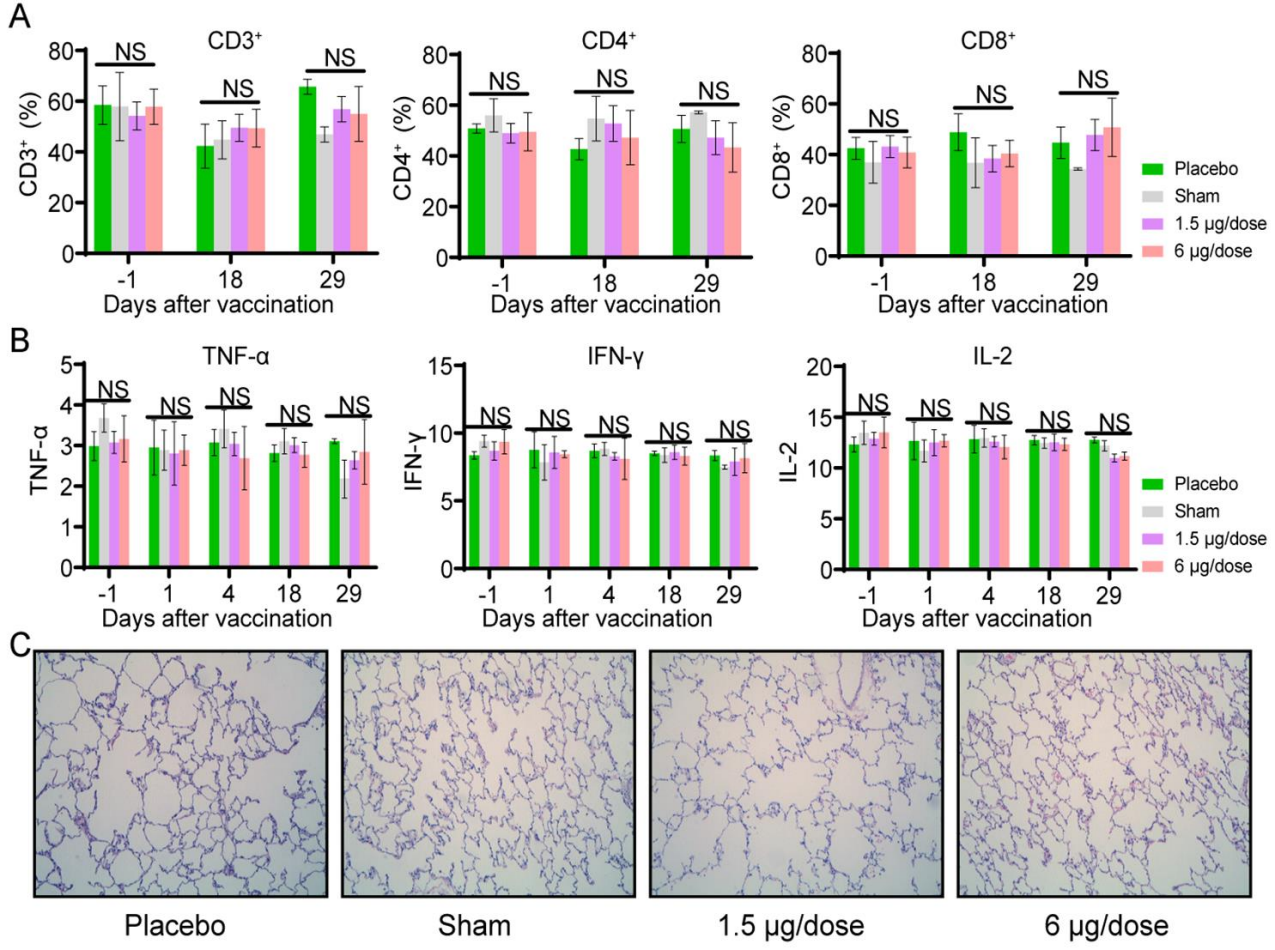
Safety evaluation of PiCoVacc in nonhuman primates. Macaques were immunized three times at day 0, 7 and 14 through the intramuscular route with low dose (1.5 μg per dose) or high dose (6 μg per dose) of PiCoVacc or adjuvant only (sham) or placebo. (**A** and **B**) Hematological analysis in all four groups of macaques (n=4). Lymphocyte subset percents (**A**), including CD3^+^, CD4^+^ and CD8^+^ were monitored at day -1 (1 day before vaccination), 18 (3 days after the second vaccination) and 29 (7 days after the third vaccination). Key cytokines (**B**), containing TNF-α, IFN-γ and IL-2 were examined at day -1, 1 (the day of the first vaccination), 4, 18 and 29 after vaccination. Data points show mean ± SD from four independent experiments; error bars reflect SD. (**C**) Histopathological evaluations in lungs from four groups of macaques at day 29. Lung tissue was collected and stained with hematoxylin and eosin.

The serious pandemic of the current COVID19 and the precipitously increasing numbers of death worldwide necessitate the urgent development of a SARS-CoV-2 vaccine, requiring a new pandemic paradigm. The safety and efficacy are essential for vaccine development at both stages of preclinical studies and clinical trials. Although it’s still too early to define the best animal model for studying SARS-CoV-2 infections, rhesus macaques that mimic COVID-19-like symptoms after SARS-CoV-2 infection appear promising animal models for studying the disease. We provide evidences for the safety of PiCoVacc in macaques; and did not observe infection enhancement or immunopathological exacerbation in our studies. Our data also demonstrate a complete protection against SARS-CoV-2 challenge with a 6μg per dose of PiCoVacc in macaques. Collectively these results suggest a path forward for clinical development of SARS-CoV-2 vaccines for use in humans. Phases I, II and III clinical trials with PiCoVacc, as well as other SARS-CoV-2 vaccine candidates, are expected to begin later this year.

## SUPPLEMENTARY MATERIALS

science.sciencemag.org/cgi/content/full/science.abc1932/DC1 Materials and Methods Figs. S1 to S6 Tables S1 and S2 References (*20*–*23*)
